# Cattle tick-borne diseases: study of knowledge and practices among communal farmers in selected areas of the Limpopo Province, South Africa

**DOI:** 10.1007/s00436-025-08529-6

**Published:** 2025-07-15

**Authors:** Realeboga Masego Gaorekwe, Maphuti Betty Ledwaba, Rae Marvin Smith, Dikeledi Petunia Malatji

**Affiliations:** 1 Department of Agriculture and Animal Health, University of South Africa, Private Bag 06Roodepoort 1710, Florida, South Africa; 2 Department of Life and Consumer Science, University of South Africa, Private Bag 06Roodepoort 1710, Florida, South Africa

**Keywords:** Knowledge and practices, Beef cattle, Farmers, Tick-borne diseases, Management practices

## Abstract

Tick-borne diseases (TBDs) pose significant challenges to livestock farmers, particularly in subtropical and tropical regions. This study aimed to assess the knowledge and practices of community cattle farmers on TBDs in Bela-Bela, Limpopo Province, South Africa. A semi-structured questionnaire was administered to 50 farmers, focused on socio-demographic information, TBD knowledge, and livestock management methods. The study found that while 78% of farmers were aware of heartwater and redwater, only 60% had sufficient knowledge to treat these diseases successfully. Terramycin and Swamycin (oxytetracycline hydrochloride) were the most used treatments, although most farmers were unaware of appropriate treatment methods for redwater. In addition to these conventional treatments, some farmers employed Ultravec as an alternative therapeutic option to manage redwater in their livestock. There was a lack of knowledge about tick species and effective management practices, and most farmers did not implement biosecurity measures to regulate livestock movements or reduce the spread of infection. The survey found that most farmers practiced extensive farming and grazed livestock mainly on communal land. Seasonal variations in disease outbreaks have been observed, highlighting the need for improved education and training on TBD management, biosecurity measures, and tick control approaches. The results indicate a significant (P ≤ 0.05) association between gender and the number of cattle reared. However, there was no significant association between farmers’ demographics and knowledge of TBDs. Strengthening farmers' understanding of disease prevention and improving access to veterinary services are critical to reducing the economic burden of TBDs in community farming systems. In addition, the use of indigenous cattle breeds with inherent tick and disease resistance is vital.

**Supplementary Information:**

The online version contains supplementary material available at 10.1007/s00436-025-08529-6.

## Introduction

In 2020, South Africa reported an estimated 12.3 million cattle comprising of diverse dairy and beef breeds (DALRRD 2021). According to the DALRRD (2021) report, primary agriculture production contributed slightly more than 2.3 percent of South Africa's GDP in 2019/20, with an estimated GDP contribution of R 81,337 million ($ 4 410 015,34), while its total gross value for 2019/20 was R 323,953 million ($ 17 565 962,68) (van Niekerk et al. 2022). Primary agricultural production describes the cultivation of crops and cattle production for food, fiber, and other basic products (DALRRD 2021). Cattle production in South Africa employs various systems such as intensive, semi-extensive, and extensive farming methods. These systems play a significant role in supporting local communities by bolstering socio-economic conditions, ensuring food security, and supporting household livelihoods (Visser et al. 2020; Van Marle-Köster et al. 2021). Indigenous cattle breeds, such as *Bos taurus* africanus (e.g., Nguni, Tuli, and Tswana) and *Bos indicus* (e.g., Zebu), are well-adapted to local conditions and exhibit resilience to adverse environments, endemic diseases, and parasites, reflecting their hardiness (Mapiye et al. 2019).

The livestock industry faces substantial economic losses due to diseases and parasitism, primarily attributable to mortality, reduced production, and diminished working efficiency (Kasaija et al. 2021). Furthermore, it has been reported that livestock farmers in tropical and subtropical areas face significant challenges due to the economic effects of ticks and tick-borne diseases (TTBDs) on livestock productivity (van den Heever et al. 2022). Tick control methods, such as chemical acaricide, have not been widely effective due to factors such as resistance, chemical residues, and associated costs (Cardoso et al. 2021). Commonly, cattle apicomplexans are managed by controlling their vectors with chemical acaricides (MacGregor et al. 2021) and treatment with narrow-spectrum antiprotozoal drugs in case of any infection. However, this method is impractical in rural areas due to resource limitations (MacGregor et al. 2021). Production losses due to tick-borne diseases are particularly severe in *Bos taurus* cattle breeds and their crosses, but *Bos indicus* cattle are also affected, especially calves and adults in a state of endemic instability. Endemic instability occurs when a disease remains present in a population, but the natural balance between the pathogen, the animals'immunity, and environmental factors is disrupted. This disruption leads to more frequent and severe outbreaks, resulting in higher mortality rates, reduced productivity, and increased costs for livestock producers (Kasaija et al. 2021).

Tick management practices are influenced by various factors such as socioeconomic level, access to veterinary care, and cultural beliefs (Namgyal et al. 2021). Ticks and tick-borne diseases (TTBDs) pose a serious threat to South Africa's agricultural economy, with the three economically important tick-borne diseases being heartwater, redwater, and anaplasmosis. In Southern Africa, cattle apicomplexans of veterinary and economic significance include *Babesia bigemina*, *Babesia bovis*, and *Theileria parva*. *B. bigemina* and *B. bovis* are causal agents of redwater, while *T. parva* causes theileriosis (MacGregor et al. 2021). According to Mtshali et al. (2004) and Choopa (2015), the major impediments to increasing cattle production and health in South Africa are anaplasmosis, redwater, and heartwater. Anaplasmosis is a tropical and subtropical disease caused by obligate intraerythrocytic rickettsiae of the genus *Anaplasma* (Jabbar et al. 2015) and is transmitted by ticks of the genus *Ixodes.* The disease presents symptoms like pyrexia, anemia, jaundice, anorexia, depression, reduced milk production, abortion in pregnant animals, and death are among the main clinical symptoms, especially in exotic breeds (Asif et al. 2022). Ticks of the genus *Amblyomma* transmit the rickettsial pathogen *Ehrlichia (Cowdria) ruminantium*, which causes heartwater (Marufu 2008). Animals with the disease display nervous symptoms as well as pericardial and pleural effusion (Kasaija et al. 2021). Redwater is caused by intraerythrocytic protozoan parasites of the genus *Babesia,* presenting acute disease symptoms such as hemoglobinuria, fever, high parasitemia, anemia, and occasionally mortality (Bock et al. 2004). The parasite is transmitted by ticks of the genus *Rhipicephalus,* which include *R. decoloratus* and *microplus* (Jabbar et al. 2015; Kasaija et al. 2021). Another TBD, Theileriosis is caused by intracellular protozoan parasites of the genus *Theileria,* which are also transmitted by ixodid ticks. Symptoms of the disease include lymph node enlargement, fever, and anorexia, which lead to lymphadenopathy and death (Magona et al. 2008). Therefore, understanding farmers'knowledge, attitudes, and practices (KAP) concerning TTBDs is imperative for designing effective control and management approaches. However, the knowledge, attitudes, and practices of communal farmers in the selected areas regarding livestock diseases are inadequately understood. Therefore, the study aims to understand the knowledge, attitudes, and practices of communal farmers in Bela-Bela, Limpopo Province regarding TTBDs in cattle.

## Materials and methods

### Study area and sample size

**Fig. 1:**
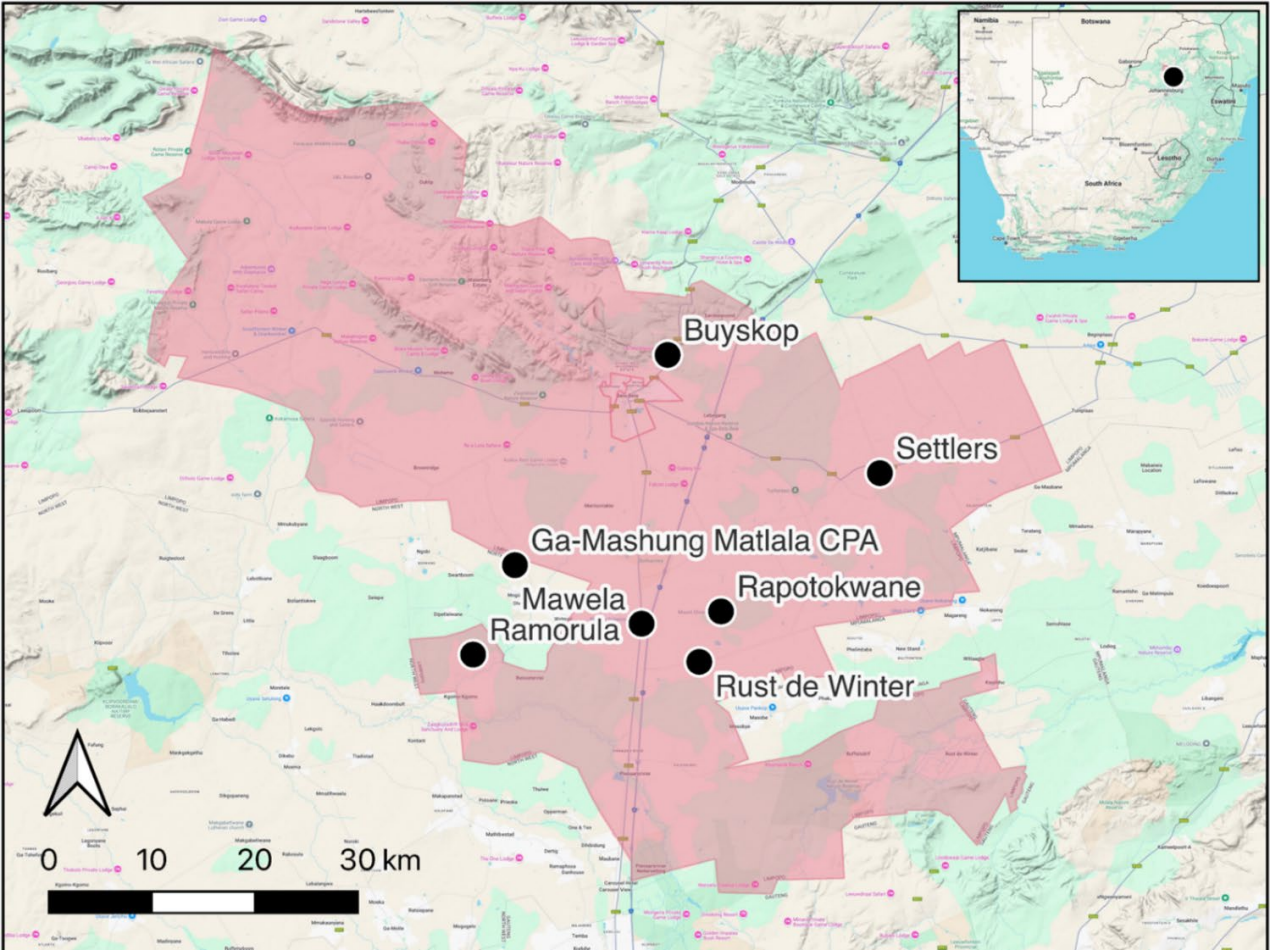
Map of Bela-Bela Municipality showing the study sites marked in black

The study was conducted in Bela-Bela, a small town geographically located within Bela-Bela Local Municipality in the Waterberg District of the Limpopo Province, South Africa (GPS coordinates: 24.8970° S, 28.2527° E). It is known for its hot mineral springs and subtropical climate and is located at the border of the North-West, Gauteng, and Mpumalanga Provinces. The warm, humid climate fosters the proliferation and distribution of ticks and contributes to the prevalence of TTBDs, affecting both livestock and wildlife populations (Statistics South Africa 2011). According to Statistics South Africa (2011), the total population in Bela-Bela is 64, 306, and a total of 2,206 households are involved in agriculture, and 696 households participate in livestock farming. The survey targeted three study areas (Fig. 1): Rust de Winter, Settlers, and Radium. From these areas, a total of 50 communal cattle farmers were selected based on their willingness to participate in the study, availability of cattle, farmers’ location, and accessibility. Farmers who participated were from seven villages: Mawela (Radium) (n = 13), Buyskop (n = 12), Raphotokwane (n = 9), Rust de Winter (n = 6), GaMashung-Matlala (n = 6), Ramorula (n = 3), and Settlers (n = 1).

### Study design and questionnaire survey

Farmers were randomly selected from a list of cattle owners maintained by the agricultural extension officers of the local Limpopo Department of Agriculture in Bela-Bela. A semi-structured questionnaire comprising of 29 items, including both open and closed-ended questions. The questionnaire was divided into three sections: section one focused on the farmers’ socio-demographic information; section two probed farmers on their knowledge and awareness of TBDs in cattle, and section three consisted of questions on biosecurity practices. Respondents aged 18 years and older, including cattle owners, herdsmen, and farm managers, were interviewed for the study. Variables collected included herd size and composition, management practices, biosecurity measures, health management, the incidence of diseases such as heartwater and redwater, vaccination, treatment, and access to agricultural extension services. In-person interviews were conducted individually and in groups in a language of the farmers’ choice at the central location, such as a local community hall and communal dip tanks for easy access to herdsmen or cattle owners.

### Statistical analysis

The data was captured on a Microsoft Excel 2010 spreadsheet and analyzed using the Statistical Package for Social Sciences (SPSS version 28). Descriptive statistics were performed using means, frequencies, and percentages to analyze questionnaire-derived variables such as socio-demographic information, production systems, herd size and composition, biosecurity, diseases, treatment, vaccination, and access to agricultural services. Knowledge and practice were converted into a binary outcome variable through scoring of the farmers’ responses by coding the questionnaire (i.e. 1 for respondents who had “adequate knowledge” and 0 for respondents who had “inadequate knowledge”), and based on the score, all the variables were converted into a binary outcome variable (i.e. 1 or 0 against each variable). Explanatory variables included age and the number of cattle per household. Based on the score, the association of respondents’ social demographic characteristics, farming practices, and knowledge of TBDs was determined using the Chi-square test and presented in tables and graphs. Statistically significant associations were determined at *P* ≤ 0.05, and the Pearson correlation test was employed to identify associations in the sample demographics.

## Results

### Socio-demographic information of respondents

**Fig. 2:**
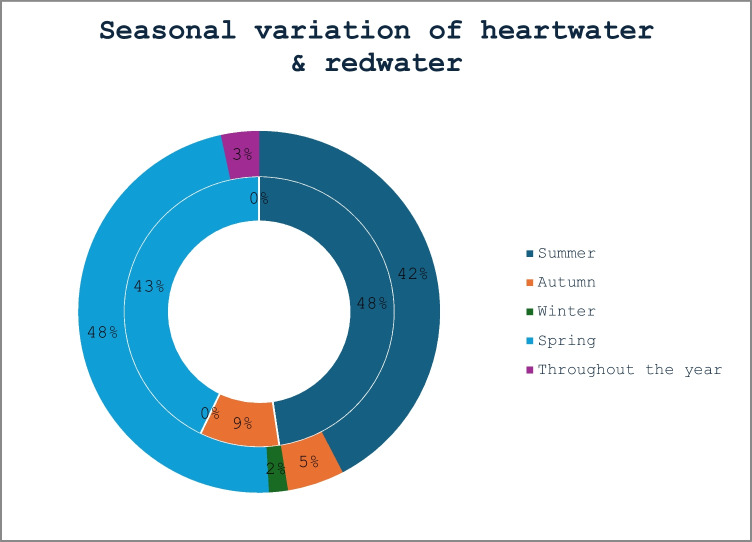
Seasonal variation in farmer-reported suspected cases of heartwater (outer circle) and bovine redwater (inner circle) diseases

A total of 50 communal cattle farmers participated in the survey (Table 1). The majority of respondents were male (74%), compared to 26% female farmers (Fig. 2). Most respondents were aged over 61 years (40%), followed by those aged 51–60 years (22%). Fewer respondents were in the 31–40-year age group (14%) and 41–50-year age group (12%), with the least represented in the 21–30-year age range (8%). Over 78% of the farmers owned fewer than 20 cattle, while 10% owned between 21–40 cattle, and only six farmers (12%) owned more than 41 cattle. Twenty-six respondents also reported keeping other livestock species on their farms or communal land: 52% reared goats, 38% kept sheep, and 24% had chickens. Notably, none of the farmers raised pigs, and a small percentage owned pets such as dogs (4%) and cats (2%). The primary reason for rearing cattle among the respondents was income (84%), followed by household consumption (36%), investment (32%), and cultural purposes (32%). Breeding (18%) and manure production (8%) were the least frequently reported reasons for cattle husbandry.

**Table 1:** Socio-demography of cattle farmers in Bela-Bela municipality, Limpopo Province

Farmers demographic information		
Items	Frequency (*n* = 50)	Proportion (%)
No. of cattle per household		
≤ 20	39	78
21–40	5	10
≥ 41	6	12
Total		100
No. of other livestock per household		
Chickens	12	24
Goat	26	52
Sheep	19	38
Pigs	2	4
Ostrich	0	0
Dogs	2	4
Cats	1	2
Donkey/Mule	1	2
Total		100
Purpose of farming		
Household consumption	18	36
Income	42	84
Source of manure	4	8
Draft purposes	0	0
Breeding	9	18
Investment	16	32
Cultural purposes	16	32
Other	2	4
Total		100
Farming practices		
Extensive	40	80
Semi-intensive	7	14
Intensive	0	0
No response	3	6
Total		100

### Livestock management employed by the respondents

In terms of livestock management, 80% of respondents utilized extensive farming systems, allowing cattle to graze freely over large areas with minimal human intervention. Conversely, 14% employed semi-intensive systems, which combine periods of grazing with supplemental feeding and limited confinement, while none practiced intensive farming, a system characterized by confinement and controlled feeding. Moreover, the majority (72%) of the farmers employed herders for cattle management, whereas 24% of the farmers cared for their cattle, and 4% relied on farm managers or relatives. All respondents allow their cattle to graze, and 2% of the interviewed farmers allow their cattle to graze with other livestock on the farm or communal land. Sheep (36%) and goats (38%) were the most mentioned livestock that graze with the respondents’ cattle, followed by donkey (2%). Twenty-eight percent of the respondents do not allow their cattle to graze with other livestock, while 2% were uncertain.

Thirty-eight (86%) of the respondents supplement their cattle feed, with the most common feed supplement being bale hay (30%), dried grasses or legumes, molasses (22%), a carbohydrate-rich byproduct of sugar production, winter licks (22%), which usually contains salt, urea, and minerals to support cattle during the colder months, and salt blocks (16%), which are primarily made of sodium chloride with trace minerals, were the most often used feed supplements. Fewer respondents reported utilizing summer licks (8%), which are similar to winter licks but made for warmer weather, finisher (6%), a high-energy supplement meant to promote weight gain, pellets (10%), which are frequently made of compressed grains, protein meals, and vitamins, and lucerne (12%), a high-protein legume forage. Other respondents also indicated utilization of SP50 (2%), a protein supplement that comprises various protein sources and soybean meal, multimeal (2%), which is a combination of minerals, oilcake, and grains, chop (2%), which respondents defined as a combination of mealie (maize), lucerne, and vitamin B2, and voerdokter (2%), a commercial supplement that contains a variety of vitamins, minerals, and protein. Of the respondents, only 10% utilized feed supplements, but did not specify the type or name.

Record-keeping practices were observed among 72% for general health and production, while 14% reported having no records. The records kept include vaccination (46%) and diseases (26%), records of causes of mortality (18%), birth rate (12%), treatments (12%), daily feed intake (14%), and the type of feed (10%). Of the 50 respondents, only three (6%) keep all records of cattle health and production.

### Awareness and seasonal patterns of tick-borne diseases

A total of 39 (78%) respondents indicated that they have heard of tick-borne diseases (TBDs), whereas 11 (22%) have not. In addition, only one participant did not respond to the question in the survey. Heartwater disease (*Ehrlichia ruminantium*) was observed more frequently during the summer and spring seasons than in winter and autumn, and only 3% of recorded cases of heartwater disease occurred throughout the year (Fig. 2). Redwater (babesiosis) disease was recorded mainly during spring (18%) and summer (10%) than autumn (4%) seasons, and there was no record of any redwater disease cases during winter. It should be noted that these reports are not based on diagnostic confirmation, but rather on producer observations.

**Fig. 3:**
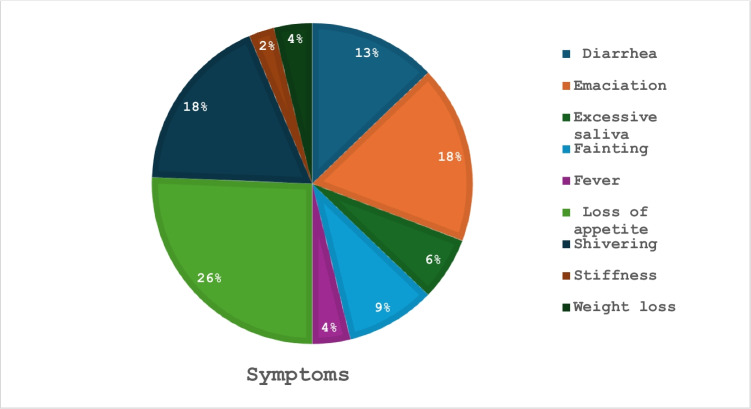
Symptoms observed by participants and recorded in association with heartwater and bovine redwater (Babesiosis) diseases

In total, 74% of respondents could identify clinical symptoms associated with heartwater and redwater, with the most recognized symptoms including loss of appetite, emaciation, and shivering at 26%, 18%, and 18%, respectively (Fig. 3). However, 26% were unable to identify clinical symptoms of both diseases, and 18 (36%) respondents indicated that they could identify clinical symptoms in other livestock. A majority of respondents (88%) were unaware of other TBDs that affect cattle, with only 14% of the respondents being aware and knowledgeable about other TBDs.

Treatment practices in this study revealed that Terramycin and Swamycin (oxytetracycline hydrochloride) (20%) were predominantly used for sick cattle. The least used treatments reported by respondents include Maxitet (4%), Hitet (4%), Ivomec (Ivermectin) (2%), and Tetracycline (2%). One respondent reported purchasing treatment medication from SWAVET, an animal health company that supplies a variety of veterinary products, including Swamycin (oxytetracycline HCl) and Swamycin LA (oxytetracycline dehydrate), which are used to treat diseases such as heartwater, anaplasmosis, and pneumonia. Another respondent used Triatix to control ticks in cattle, while another used Ultravac to treat redwater disease. Other respondents (40%) did not know how to treat sick animals. Only 10% of the respondents indicated that state animal health technicians and veterinarians treat their sick animals. Most respondents reported treating their cattle for heartwater occasionally, some treat fortnightly, while other respondents treat when they show clinical signs, and only four respondents occasionally treat cattle for redwater.

The majority (66%) of the respondents indicated that access to their farm is not restricted, and 76% do not have biosecurity measures for visitors in place. Non-restriction alludes to access to the farm by anyone at any time. Twenty-one (42%) surveyed respondents have records of livestock movements on their property, whereas 58% do not keep any records of animal movement. Although several respondents herd cattle within proximity, 54% of them do not discuss treatment and control measures for heartwater and redwater outbreaks with neighboring farmers. Thirty-four (68%) respondents regularly discuss acaricide treatment against ticks with state animal health technicians or state veterinarians.

### Association of farmers’ demographic information and knowledge of tick-borne diseases

There was no association between farmers'demographic information and knowledge of TBDs (P ≥ 0.05). However, age and gender were found to contribute to the number of cattle and other livestock kept on the farm. The results also indicate that there is a significant association (P ≤ 0.05) between gender and the number of cattle kept by farmers. Pearson correlation test reveals that there is no significant association between farmers’ age and the number of cattle. In addition, a chi-square test was performed, and no significant correlation (P ≥ 0.05) was observed between variables such as farmer gender, age, and knowledge of TBDs.

## Discussion

This study presents the knowledge and practices of communal cattle farmers in the Southern region of the Limpopo Province regarding TBDs. The findings revealed that the majority of cattle owners surveyed were males, which is in line with the general trend of enduring male dominance in the cattle farming sector. These results corroborate with findings by Lehloenya et al. (2007), Katiyatiya et al. (2014), Yawa et al. (2020), Olaogun et al. (2023), Monkwe et al. (2023), and Malatji & Antwi (2024) who reported that managerial challenges such as the physical handling of animals, and lifting and carrying heavy objects, contribute to the continued dominance of males in the sector. Similar findings were also reported in Zimbabwe (Ndhlovu and Masika 2013) and Nigeria (Nnabuife et al. 2023). Cultural norms and social structures contribute to the unequal gender distribution in cattle farming, resulting in more male presence than females (Njenga and Gurung 2019). Therefore, stakeholders engaged in policy development should examine and evaluate disparities in existing agricultural policies and identify practical policy measures to address these gaps.

The majority of the farmers were aged 61 and above. Monkwe et al. (2023) also noted this, suggesting that elderly farmers have more time to farm than young individuals who are either studying towards other professions or are employed in cities. Being elderly, most of these farmers hire herdsmen to care for their cattle, and this is similar to findings by Habiyaremye et al. (2017) and Malatji and Antwi (2024). The findings further revealed that farmers primarily allowed their cattle to graze in the open veld, which is consistent with findings by Monkwe et al. (2023), who reported that communal farmers in Limpopo typically release their cattle for grazing in the morning and return them home to camp at night. The authors mentioned above also noted that some farmers rely on communal water and grazing lands as the respective sources of drinking water and feed, which often results in minimal provision upon the animals’ return to the camps. This practice can be linked to limited resources and capital. While extensive farming allows for lower input use, making management more feasible, this approach contributes to overgrazing and overstocking, possibly increasing the risk of tick exposure, and limiting the availability of palatable grass.

In this study, farmers reported encountering TBDs primarily during the summer and spring seasons, and these findings corroborate with results by Yawa et al. (2020), who reported a high prevalence of TBDs during the summer season. Previous studies (Marufu et al. 2010; Katiyatiya et al. 2014) also observed ticks and TBDs during the wet (rainy) season. Furthermore, Monakale et al. (2024) documented that the occurrence of ticks in the Limpopo Province was most frequently recorded during summer. High tick loads can severely impact cattle production and fertility, as well as decrease milk yield (Perera et al. 2014), and in extreme cases, can result in mortality (Moumouni et al. 2023). Climate and other environmental changes are expected to exacerbate the risk of TTBDs in a variety of ways, including the increase in the range of animal reservoir hosts (Bouchard et al. 2019).

In the current study, most farmers were able to identify clinical symptoms of heartwater and redwater. According to Bekker et al. (2001) and Allsopp (2015), the common symptoms of heartwater include convulsions, hanging head, fever, depression, heavy breathing, exaggerated blinking, loss of appetite, anorexia, and mortality, while redwater typically presents symptoms such anemia, fever, decreased milk production, hemoglobinuria, nervous signs, weakness and occasional mortality (Al-Hosary (2017). Study participants reported observing red urine, which is a characteristic of redwater disease. Literature reports revealed that fever is the most severe symptom for heartwater, which is consistent with findings reported by Chenyambuga et al. (2010). However, only three respondents identified this clinical symptom in their cattle, suggesting that some individuals may have been unable to recognize the presence of fever due to limited access to tools such as thermometers for temperature checks, mistakenly regarding it as excessive shivering.

Communal cattle grazing increases exposure to multiple tick species that are vectors of tick-borne pathogens (TBPs), including heartwater, babesiosis, and anaplasmosis, which can subsequently be transmitted from one host to another (Moutailler et al. 2016). The epidemiology of TBDs is quite complex, demonstrating the variation of tick species with different life cycles and modes of transmission (Ostfeld et al. 2006). *Babesia* species, a causative agent of redwater, can be transmitted both transovarially and transstadially, by *Rhepicephalus* tick species (Chauvin et al. 2009; Madder et al. 2013), while heartwater is primarily transmitted by *Amblyomma hebraeum,* a three-host species that is known to transmit pathogens transstadially (Madder et al. 2013). While rotational grazing and acaricide application can interrupt the tick life cycle and reduce tick burden, the effectiveness of this method depends on proper implementation and the biology of ticks (Chauvin et al. 2009). Moreover, the aggravating difficulties of controlling these diseases are the financial limitations experienced by farmers, including inadequate financial resources to purchase acaricides or drugs to treat TBDs.

In this study, most farmers used Terramycin and Swamycin (oxytetracycline hydrochloride) to treat animals with heartwater. However, most farmers had little knowledge of the treatment of redwater. Chenyambuga et al. (2010) reported that farmers utilized oxytetracycline to treat redwater and heartwater, and diminazene to treat redwater. Interestingly, while the participants in this study are aware of these diseases and their clinical symptoms, they lack knowledge about the ineffectiveness of antibiotics in treating protozoan infections. Therefore, it is imperative for the Department of Agriculture, Land Reform and Rural Development (DALRRD, 2021) to improve the knowledge and practices of smallholder farmers by developing and enforcing actionable policies to improve surveillance, control, and reduce the impact of TTBDs in rural communities. Moreover, developing veterinary strategies to assist farmers, especially those in remote areas, is crucial as it will aid farmers in disease diagnosis, treatment, and vaccination. It is also imperative for farmers to implement tick control measures using acaricides to reduce tick populations. Promoting integrated and sustainable tick control methods and educating farmers on regular monitoring of ticks for timely intervention is imperative to prevent outbreaks of TBDs. The DALRRD could also educate farmers on the importance of rotational grazing, especially in regions with the highest tick infestation, to help break the life cycle of ticks and reduce the risk of disease transmission. The use of vaccines can aid farmers a great deal in reducing the incidence and severity of infections. Although farmers already provide feed supplements, emphasis on the importance of a well-balanced diet for their cattle is necessary to improve cattle immunity and resilience against TTBDs. In addition, emphasis on community-based initiatives is key to improving the dissemination of information and collaboration in tick management among farmers to reduce and prevent TTBDS, especially among communal or neighbouring farmers.

This study revealed that many farmers had no biosecurity measures in place and lacked control measures for animal movements. These findings are consistent with a report by Horak et al. (2011), which highlights the potential exposure of cattle in communal areas to wildlife, potentially leading to exposure to TBDs. Several tick species infest one to three hosts, increasing exposure to tick-borne pathogens, and persistent use of acaricides could result in resistance in ticks. Although most farmers were familiar with certain TBDs, such as heartwater and redwater, they lacked knowledge about the control of these diseases. Furthermore, the data indicate a lack of knowledge about other TBDs affecting cattle. Congruent with a report by Namgyal et al. (2021), deficiencies in awareness programs on TBDs in communal areas are possible, likely due to limited resources. Additionally, this might also be attributed to the lack of interest in participating in events organized by the DALRRD hence, the need to encourage farmers'participation in events organized by the DALRRD to enhance their knowledge, attitudes, and practices regarding TBDs. Therefore, based on our findings, we recommend that the local DALRRD improve the scope of their education and training programs for smallholder farmers on TTBDs by hosting events tailored to the needs of the local community and conducting them in the native language for better understanding.

## Conclusion

The present study identified the farmers’ knowledge and practices on TBDs in communal areas. This is an indication that some cattle owners are knowledgeable about controlling and mitigating the impact of TTBDs. However, regular workshops by the public and private sectors are crucial to further educate communal farmers on TBDs. Databases are important to enable farmers to access information about tick species and TBDs and find sustainable, cost-effective measures to control and prevent future outbreaks of TBDs. Strict biosecurity measures must be considered before receiving or selling cattle to reduce the spread of infection.

## Supplementary Information

Below is the link to the electronic supplementary material.

Supplementary file1 (XLSX 24.6 KB)

## Data Availability

No datasets were generated or analysed during the current study.
